# Facile Synthesis of Sustainable Tannin/Sodium Alginate Composite Hydrogel Beads for Efficient Removal of Methylene Blue

**DOI:** 10.3390/gels8080486

**Published:** 2022-08-03

**Authors:** Jie Gao, Zhenzhen Li, Ziwen Wang, Tao Chen, Guowen Hu, Yuan Zhao, Xiaobing Han

**Affiliations:** Hubei Key Laboratory of Radiation Chemistry and Functional Materials, School of Nuclear Technology and Chemistry & Biology, Hubei University of Science and Technology, Xianning 437100, China; gaojie2019@hbust.edu.cn (J.G.); l3373348038@126.com (Z.L.); a13872354284@163.com (Z.W.); hgwpublic@163.com (G.H.); zhyf308@hbust.edu.cn (Y.Z.)

**Keywords:** adsorption, hydrogel beads, tannin, sodium alginate, methylene blue

## Abstract

To meet the requirement of sustainable development, bio-based adsorbents were developed for the removal of dye contaminant. To improve the adsorption capacity of pure sodium alginate (SA) adsorbent for the removal of methylene blue (MB), aromatic bio-based tannin (Tan) was incorporated through the cross-linking with calcium ion. The obtained Tan/SA composite hydrogel beads were characterized with SEM, FTIR and TG, demonstrating that millimeter-sized beads were obtained through calcium cross-linking with enhanced thermal stability. The maximum capacity (247.2 mg/g) at optimal condition (pH = 12, T = 45 °C) was obtained for the 40%Tan/SA adsorbents, with a removal efficiency of 82.4%. This can be ascribed to the electrostatic attraction between SA and MB, as well as the formation of π–π stacking between Tan and MB. The adsorption process for MB is endothermic, and chemical adsorption, the removal efficiency was exceeded 90% after five cycles.

## 1. Introduction

Synthetic dyes have been widely used in textile industries, causing serious pollution in water resources. Once the synthetic dyes enter the human body, it can endanger people’s health and cause a variety of diseases [1,2]. The synthetic dyes are classified into three types, namely cationic dye, anionic dye and non-ionic dye, according to the charge of the molecules. Among these dyes, methylene blue (MB) is a typical cationic dye which has been widely used in the field of textile, paper, coating and printing. It can easily combine with oppositely charged cell membranes, leading to a high hazard for organisms. In addition, the MB molecules possess a very high color value, which can be clearly observed even at relatively low concentrations (<1 mg/L) [3,4]. Therefore, the removal of MB from wastewater is an important issue.

To meet the requirements of sustainable development, many bio-based adsorbents, especially for composite hydrogel were fabricated for the adsorption of MB in wastewater. Activated lignin-chitosan extruded pellets were reported for the removal of MB, the adsorption results fitted well with the Langmuir isotherm, while the maximum adsorption capacity is 36.25 mg/g [5]. Chitosan/sepiolite composite hydrogel were prepared with cross-linking using epichlorohydrin, the CS50SP50 was selected as the best adsorbent for the adsorption of MB, and the monolayer adsorption capacities of this composite is 40.986 mg/g [6]. Sodium alginate/soybean extract composite hydrogel beads were reported for the removal of MB by Viscusi et al. [7], the adsorption data can be described with a second-order model, and the maximum adsorption capacity is 49 mg/g. Lu et al. reported magnetic activated carbon/sodium alginate composite hydrogel beads for the adsorption of MB [8], and the maximum adsorption capacity is 222.3 mg/g with initial concentration of 700 mg/L, with a removal efficiency of 31.8%. Alginate/activated carbon composite hydrogel beads were also reported for the removal of MB [9], and a higher adsorption capacity (730 mg/L) was obtained with high initial concentration of 1000 mg/L at 40 °C, with a removal efficiency of 73%. Though different bio-based adsorbents were developed for the removal of MB, the adsorption capacity or the removal efficiency need significant improvement.

The intermolecular interaction between the adsorbents and the dye is an important factor to improve the adsorption performance. The commercial dyes are charged molecules with aromatic structures, for example, MB is a positive dibenzothiazine derivative. Based on its structure feature, many different adsorbents were designed and fabricated. Due to its positive charge of MB, electrostatic attraction was taken into account, firstly in the adsorption design. Many anionic polymers were prepared for the removal of MB, including sodium polystyrene sulfonate [10], polyacrylic acid [11] and bio-based sodium alginate [7,8,9]. As π-π stacking interaction can form between dyes and the compound containing an aromatic structure, the introduction of an aromatic structure was taken into account in the adsorbent design. In order to improve the adsorption performance, bio-based aromatic lignin [12] and tannin [13] were introduced into the polymer matrix to enhance the adsorption capacity. 

**Figure 1:**
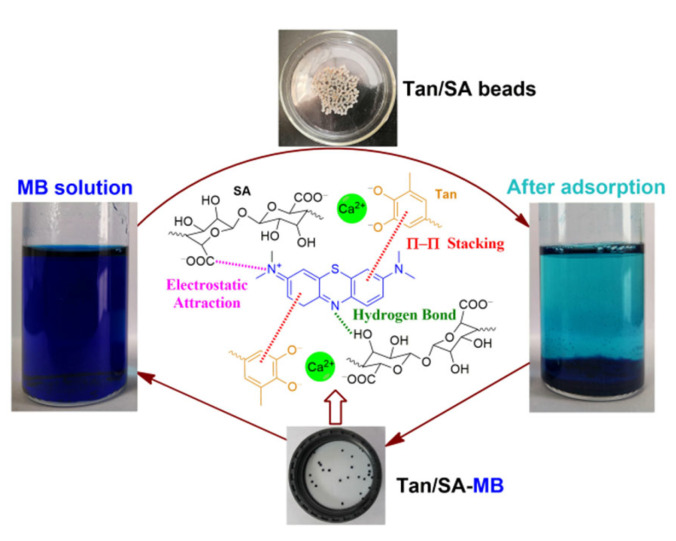
Bio-based Tan/SA hydrogel absorbent for the removal of MB.

The composites consisting of bio-based tannin (Tan) and sodium alginate (SA) can not only meet the requirement of sustainable development, but also lower the cost and extend the application of the production. Tan/SA films were reported by Kaczmarek et al. [14], and improved physicochemical properties were observed for the composite films. In addition, the films showed antioxidant properties, which have a promising application in traditional packaging material. Larosa et al. reported the characterization of Tan/SA beads by morphology, thermal stability, FTIR and XRD [15], and the non-crystalline property makes them suitable as carriers for possible controlled release, and they could be used in food applications to improve tea quality or clarify juices. The enhanced drug encapsulation and extended release profile of SA with the incorporation of Tan was reported by Abulateefeh et al. [16]; the incorporation of Tan led to a more than fourfold increase in drug encapsulation efficiency and reduced burst drug release from 44% to around 10% within the first 30 min. Though the bio-based Tan/SA composite was prepared and investigated, there was no report for the removal of MB with Tan/SA beads. Therefore, Tan/SA beads were fabricated by cross-linking with calcium ion and used for the removal of MB (Figure 1); the adsorption of MB can be realized with electrostatic attraction originated from SA and can be improved through π-π stacking interaction with incorporated aromatic Tan. The obtained Tan/SA beads were characterized with FTIR, SEM and TG, with the influence of experimental parameters, and the thermodynamic, kinetic and regeneration were also investigated.

## 2. Results and Discussion

### 2.1. Characterization of Tan/SA Beads

#### 2.1.1. Morphology Analysis

**Figure 2:**
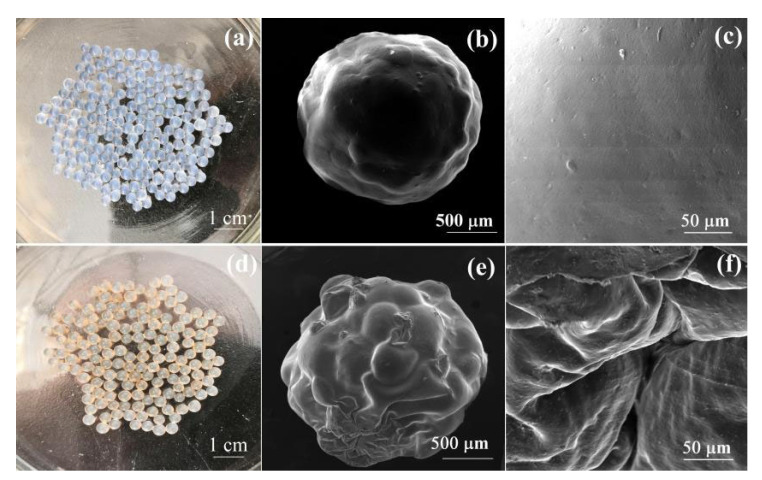
Photos of SA beads (**a**) and Tan/SA-40% beads (**d**), SEM images of SA beads (**b**,**c**), Tan/SA-40% beads (**e**,**f**).

The photos and surface morphology of pure SA beads and 40% Tan/SA beads are shown in Figure 2a–f. The pure SA beads exhibit colorless transparency, while the color changed to light yellow for the 40% Tan/SA beads. The obtained adsorbents are spherical in shape and show a uniform size distribution, with a diameter of about 2 mm. The prepared composites are millimeter-sized beads, similar to the commercial adsorption resin, which is of benefit for the application in industry [17,18]. The pure SA beads present a smooth surface, while the 40% Tan/SA beads show a rough surface with many folds. The introduction of Tan into the SA matrix enhances the surface area of the obtained adsorbents, which will improve the adsorption capacity [12].

#### 2.1.2. Structure Analysis

**Figure 3:**
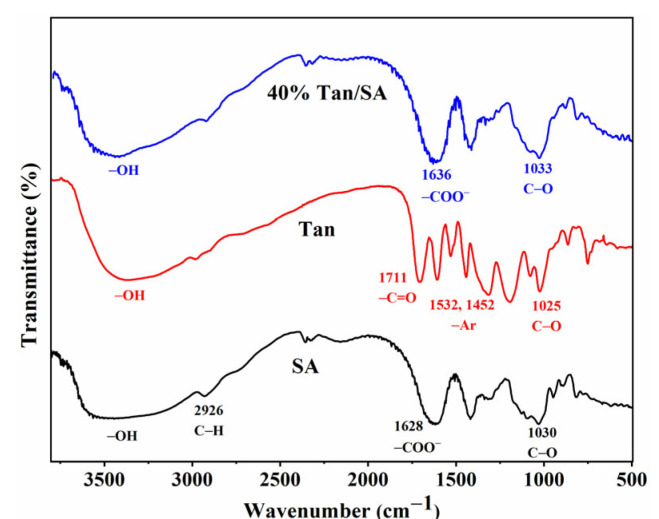
FTIR spectra of SA, Tan, 40% Tan/SA beads.

As shown in Figure 3, the structure of SA, Tan and 40% Tan/SA beads was revealed by FTIR. In the spectra of SA, the vibrations of –OH were confirmed with the broad peak from 3000 to 3600 cm^−1^, the presence of stretching vibration (2926 cm^−1^) and bending vibration (1415 cm^−1^) revealing the existence of an aliphatic C–H bond. The peak around 1628 cm^−1^ can be ascribed to the carboxylate ion, and the peaks at 1030 and 950 cm^−1^ can be assigned to the C–O vibrations [14]. The spectrum of Tan shows a broad band from 3100 to 3500 cm^−1^, which corresponds to the stretching of –OH in the phenolic groups. In addition, strong and sharp peaks at 1711 and 1608 cm^−1^ are attributed to the carbonyl stretching of ester groups and aromatic C–O stretching vibrations. The peaks at 1532 and 1452 cm^−1^ can be attributed to the C–C stretching for the aromatic skeleton [15], which is of benefit for the formation of π–π stacking with aromatic MB in the adsorption, leading to the enhancement of adsorption capacity. For the 40% Tan/SA beads, all the peaks of SA and Tan can be observed, and the peak of 950 cm^−1^ for the SA disappeared. In addition, the carbonyl stretching peak shifts from 1628 to 1636 cm^−1^, revealing the coordinate nature of calcium-bonding; thus, the Tan/SA beads were successfully fabricated with the calcium ion cross-linking [14,15].

#### 2.1.3. Thermal Stability Analysis

**Figure 4:**
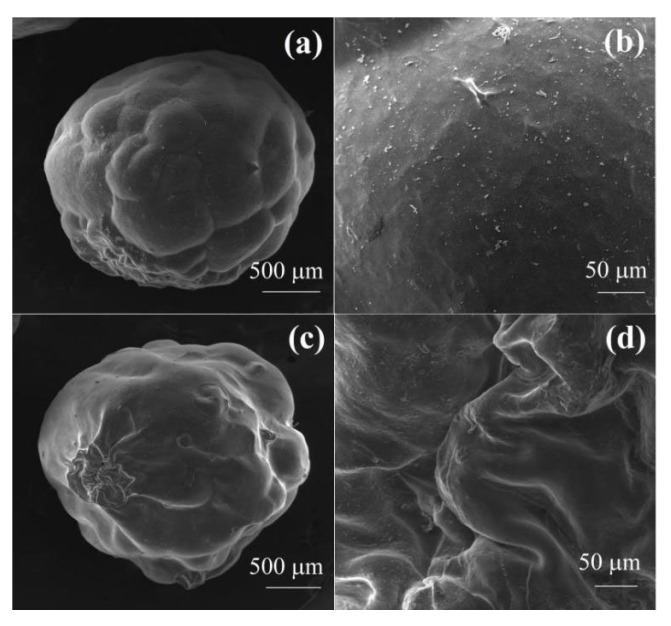
SEM images of SA hydrogel beads (**a**,**b**), Tan/SA-40% hydrogel beads (**c**,**d**) after heat treatment (T = 50 °C, t = 48 h, s = 200 rpm).

The stability of the hydrogel beads was investigated, and the SEM images of the pure SA hydrogel beads and Tan/SA-40% hydrogel beads after heat treatment is presented in Figure 4. Under the condition of 50 °C, after shaking for 48 h at 200 rpm, the morphology of pure SA hydrogel beads and Tan/SA-40% hydrogel beads showed no significant change, demonstrating that the hydrogel beads have a good stability in an aqueous solution, which will be stable under the adsorption experimental condition.

**Figure 5:**
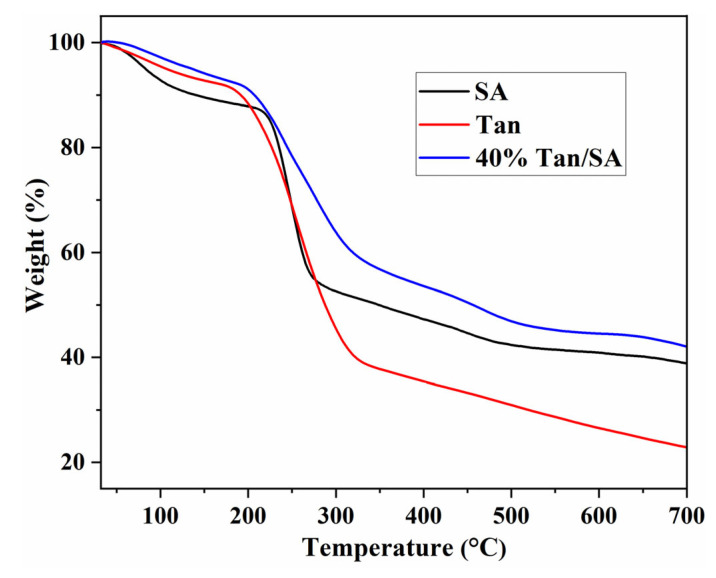
TG spectra of SA, Tan and 40% Tan/SA beads.

The TG curves of Tan, SA and 40% Tan/SA beads are shown in Figure 5. The Tan exhibits a three-stage degradation process; there is a loss of adsorbed water (about 7.5%) before 180 °C. The decomposition of linkage between flavonoid units is in the temperature range of 180–320 °C, with a weight loss of 33%. The pyrolytic degradation of flavonoid units occurs after 320 °C, and the residue char is 22.8% at 700 °C [19,20]. The TGA curves of SA gave four mass loss before 700 °C. The first stage, which is approximately 7.2% before 120 °C, is attributed to the evaporation of absorbed water. In the second stage, the mass loss of about 6.1% between 150 and 220 °C could be due to the decarboxylation of SA released carbon dioxide. The third stage mass lose between 220 and 450 °C is assigned to the thermal decomposition of the aminopropyl groups and bisaldehyde. The last stage indicates that the organic species have been completely decomposed, and the residue char is 38.6% at 700 °C [8]. The 40% Tan/SA beads showed a similar degradation behavior to pure SA, and improved thermal stability was obtained for the composite beads. This can be ascribed to the rigid structure of Tan molecules, which is more stable than the flexible chain of SA [8,12].

### 2.2. Adsorption Behavior of Tan/SA Hydrogel Beads

The MB is positive charged dye with an aromatic structure. As mentioned in the structure analysis (Figure 3), the obtained Tan/SA beads possess a carboxyl group with a negative charge and aromatic ring (Tan). The MB can be adsorbed onto the Tan/SA beads through the combination of electrostatic attraction, π-π stacking, according to the literature [10,11,12,13]. The fabricated Tan/SA beads were used for the removal of MB, under different adsorption conditions.

**Figure 6:**
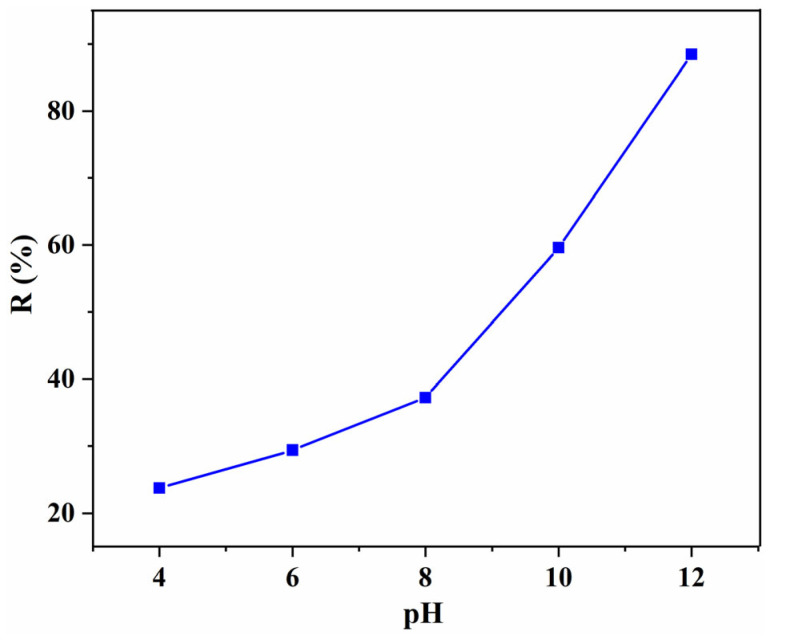
Effect of the initial pH on the removal efficiency with 40% Tan/SA beads (*C*_0_ = 80 mg/L, T = 25 °C, t = 4 h).

As shown in Figure 6, the removal efficiency for the 40% Tan/SA beads under different pH values were investigated first. The adsorption behavior of Tan/SA beads show a pH dependence toward MB, demonstrating that the electrostatic attraction plays an important role in the adsorption process. With the increase in the pH value, the removal efficiency increased obviously, and the highest R (88.46%) was observed at pH = 12. This can be ascribed to the disadvantage of high H^+^ concentration; the H^+^ has a competitive adsorption with positive MB [10]. Though a high pH value is of benefit for the enhancement of removal efficiency, taking the protection of the environment into account, adsorption at a higher pH (>12) was not conducted. 

**Figure 7:**
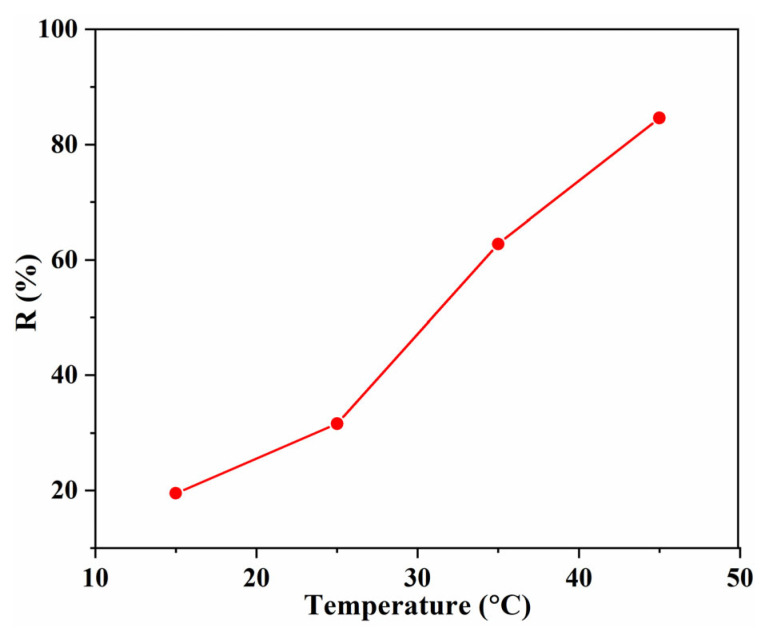
Effect of the temperature on the removal efficiency with 40% Tan/SA beads (*C*_0_ = 80 mg/L, pH = 7, t = 4 h).

The effect of temperature on the removal efficiency was also investigated (Figure 7). With the increase in the solution temperature, the removal efficiency increased dramatically, and the highest R (84.62%) was obtained at 45 °C. This phenomenon revealed that the adsorption is an endothermic process, which may be assigned to the increase in the mobility of MB molecules and the number of active sites of Tan/SA beads [21]. Though high temperature is of benefit for the enhancement of removal efficiency, taking the energy consumption into account, adsorption at a higher temperature (>45 °C) was not conducted. 

The thermodynamic parameters, including the equilibrium constant *K*^0^, the change of free energy Δ*G*^0^, entropy Δ*S*^0^ and enthalpy Δ*H*^0^ can be calculated using the following equations [12,22,23]:

*lnK^o^* = *ln*(*Q_e_*/*C_e_*) (1)

Δ*G*^0^ = −*RT ln K^o^* (2)

*Rln K^o^* = −Δ*H*^0^/*T* + Δ*S*^0^ (3)

The *Q_e_* is adsorption capacity (mg/g), *C_e_* is equilibrium concentration, *R* is the universal gas constant (8.314 J/mol·K) and *T* is the Kelvin temperature. The Δ*H*^0^ and Δ*S*^0^ are calculated from the linear plot of ln *K*^0^ versus 1/*T*.

The calculated thermodynamic parameters are listed in Table 1. With the increase in the temperature, the Δ*G*^0^ decreased gradually, illustrating that high temperature can accelerate the adsorption process, which is consistent with the results mentioned above. When the temperature is no more than 298 K, the value of Δ*G*^0^ is positive, indicating that the adsorption cannot proceed spontaneously. With the increase in temperature, the value of Δ*G*^0^ changed to negative, revealing that the adsorption can proceed spontaneously at a high temperature. The Δ*H*^0^ and Δ*S*^0^ calculated from the slope and intercept are 80.80 kJ/mol and 0.27 kJ/mol·K, respectively. The positive value of Δ*H*^0^ demonstrated an endothermic feature of the MB adsorption again, and the positive Δ*S*^0^ values showed the good affinity of Tan/SA beads for MB during the adsorption process [24,25,26].

**Figure 8:**
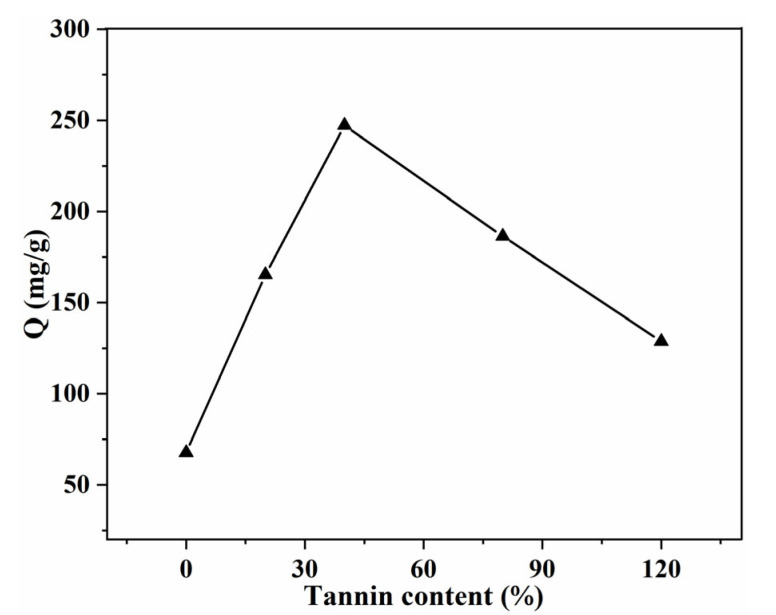
Effect of Tan content on the adsorption capacity (*C*_0_ = 200 mg/L, pH = 12, T = 45 °C, t = 24 h).

Under the optimal conditions (pH = 12, T = 45 °C), the effect of Tan content on the adsorption capacity was investigated (Figure 8). With the incorporation of Tan, the adsorption capacity of MB enhanced obviously. The highest adsorption capacity (247.2 mg/g) was observed for the 40% Tan/SA beads, with a removal efficiency of 82.4%. In comparison with pure SA beads, the maximum adsorption capacity increased by 264%. This can be ascribed to the formation of strong interaction of π-π stacking, between the aromatic tannin and the MB. With a further increase in the tannin content, the adsorption capacity decreased, which is due to the hindered effect of excessive rigid tannin for the diffusion of MB molecules [12,27].

### 2.3. Adsorption Kinetics of Tan/SA Hydrogel Beads

**Figure 9:**
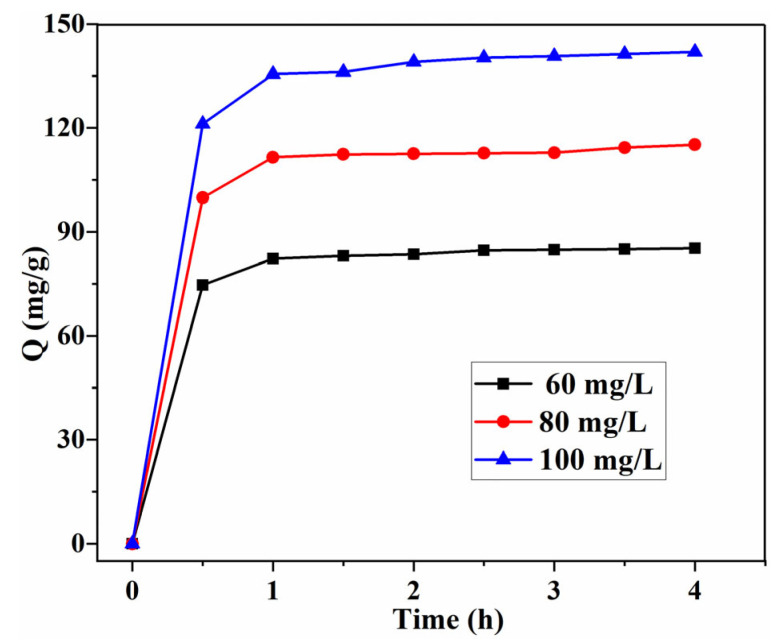
Effect of initial concentration and contact time on the adsorption capacity Q (mg/g) with 40%Tan/SA beads (pH = 12, T = 45 °C).

As shown in Figure 9, the influence of concentration with the increase in contact time (within 4 h) on adsorption was investigated. The adsorption rates are fast with different initial concentrations (60, 80, 100 mg/g); with the increase in the initial concentration of MB, the equilibrium time increased from 2 to 3 h, revealing that the adsorption of MB is dependent on the concentration. This is because it takes a long time to diffuse into the internal surface for MB molecules, after the saturation adsorption on the external surface of the Tan/SA beads [28,29].

The adsorption kinetic can reveal the adsorption efficiency and determine the potential application of the obtained bio-based Tan/SA composite adsorbents. To give a deep insight into the adsorption process and adsorption mechanism for MB adsorption onto Tan/SA beads, pseudo-first-order, pseudo-second-order and Elovich models were used to evaluate the adsorption kinetic (Equations (4)–(6)) [30,31,32,33,34]. 

Pseudo-first-order:

(4) $$\log(q_{e}-q_{t})=\log q_{e}-k_{1}t$$

Pseudo-second-order:

(5) $$\frac{t}{q_{t}}=\frac{1}{k_{2}q_{e}^{2}}+\frac{t}{q_{e}}$$

Elovich model:

(6) $$q_{t}=\frac{\ln\alpha \beta }{\beta }+\frac{1}{\beta }\ln t$$

where *q_e_* and *q_e_* are the adsorption capacity at equilibrium and at time *t*, respectively, k_1_ and k_2_ are the rate constant, α is the initial adsorption rate and β is the desorption constant.

**Figure 10:**
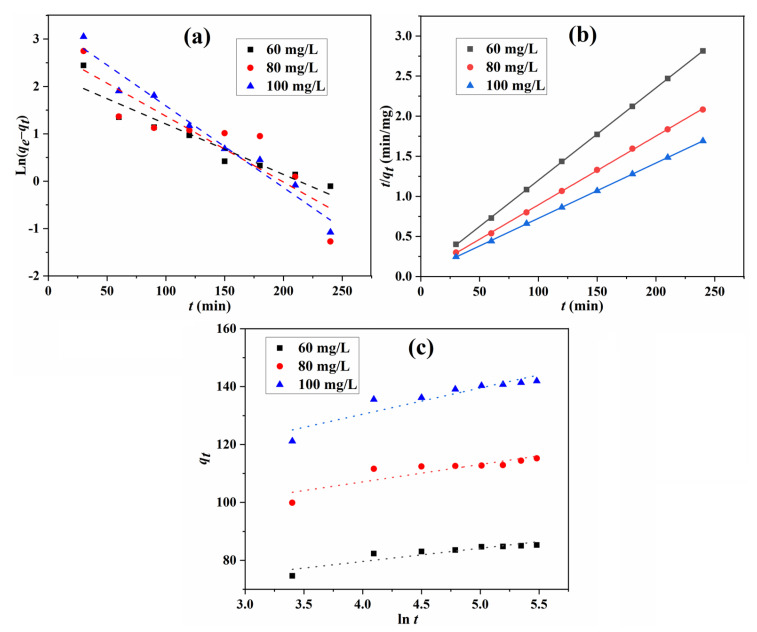
Fitting of pseudo-first-order (**a**), pseudo-second-order (**b**), and Elovich (**c**) kinetic models for the MB adsorption.

The fitting curves of MB adsorption with the three kinetic models are shown in Figure 10, and the parameters calculated from these curves are listed in Table 2. For the equilibrium adsorption capacity at different initial concentrations, the experimental value is very close to the calculated one for the pseudo-second-order model. The correlation coefficient R^2^ (0.9999) shows that this model fits the experiment data better than that of the pseudo-first-order and Elovich model, demonstrating that the pseudo-second-order is predominant in the adsorption process, and the chemical adsorption is the rate-limiting step for the MB molecules’ adsorption [10,12,35]. 

### 2.4. Regeneration Behavior of Tan/SA Hydrogel Beads

**Figure 11:**
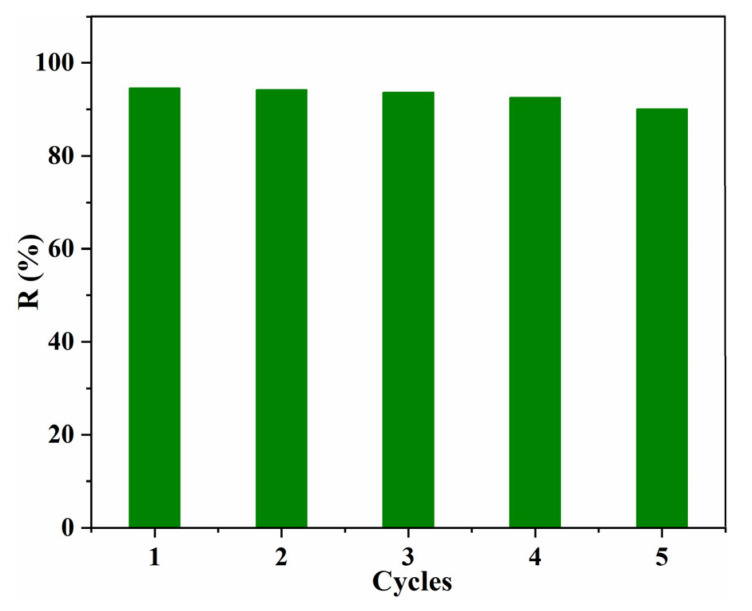
Effect of recycling times on the adsorption capacity Q (mg/g) with 40%Tan/SA beads (*C*_0._ = 10 mg/L, pH = 12, T = 45 °C, t = 4 h).

Recyclability is an important factor in evaluating the performance of the obtained bio-based Tan/SA absorbents; therefore, the prepared Tan/SA beads were evaluated by the adsorption/desorption experiment. The adsorption experiment was conducted with an MB concentration of 10 mg/L at pH = 12 and T = 45 °C for 4 h. The desorption of MB molecules from Tan/SA was achieved with 1 M HCl aqueous solution in an incubator shaker and washed with distilled water several times. As shown in Figure 11, the removal efficiency of MB decreased slightly, and the removal efficiency still exceeds 90% after five cycles. This result revealed that the adsorption ability was not changed obviously after regeneration, indicating that the Tan/SA adsorbents have a good reusability [10,28]. 

### 2.5. Comparative Analysis of MB Adsorption

The adsorption capacity, removal efficiency and regeneration of the obtained Tan/SA beads was compared with similar bio-based adsorbents in reported literature. As shown in Table 3, there was no regeneration data for most of the reported bio-based adsorbents. Some of the reported adsorbents show very high adsorption capacity at much higher initial concentrations, but the removal efficiency is relatively low. The adsorption capacity or removal efficiency of the Tan/SA beads was better than most of the similar bio-based adsorbents. The improved adsorption performance of the obtained bio-based adsorbents can be ascribed to the incorporation of aromatic Tan, which can form π-π stacking interaction with aromatic MB in the adsorption [12,13].

## 3. Conclusions

Sustainable millimeter-sized Tan/SA composite hydrogel beads were successfully synthesized for the efficient removal of MB, which will benefit environment protection, sustainable development and industrial application. The introduction of aromatic Tan into the SA matrix can not only enhance the thermal stability of the prepared beads, but also improve the adsorption performance through the formation of π-π stacking. The maximum capacity at optimal condition was obtained for the 40%Tan/SA adsorbents, with a removal efficiency of 82.4%. The positive of Δ*H*^0^ demonstrated an endothermic feature of the MB adsorption, and the calculated kinetic parameters revealed that the chemical adsorption is the rate-limiting step for the MB molecules’ adsorption. The removal efficiency of MB still exceeds 90% after five cycles, which indicated that the Tan/SA adsorbents have a good regeneration ability. The fabricated bio-based Tan/SA adsorbents have a promising application for the removal of MB in industry. Future work will focus on the selectivity, continuous column adsorption and scale-up of this kind of bio-based adsorbent.

## 4. Materials and Methods

### 4.1. Materials

All reagents were purchased from commercial suppliers of analytical grade reagent and used without further purification. Tannin (Tan, 98%, Mw = 1700 g/mol), sodium alginate (SA, 200 mPa·s), CaCl_2_ and methylene blue (MB, 98%) were purchased from HWRK chemical Co. Ltd. (Beijing, China). Distilled water was used throughout the experiments for solution preparation. 

### 4.2. Preparation of Bio-Based Tan/SA Hydrogel Beads

The bio-based Tan/SA beads were prepared as follows [14,15,16]: the Tan was dispersed into 30 mL water using a bath sonicator. Then, 0.6 g SA was added to the dispersion under stirring until complete dissolution at 60 °C, the weight percentage of Tan to SA is 0, 20, 40, 80, 120 wt%). The obtained mixture was dropwised into 5 wt% CaCl_2_ solution at room temperature without stirring, the cross-linking reaction kept for 24 h. Tan/SA composite beads were obtained with filtration, washed three times with distilled water. The obtained hydrogel beads were dried at 60 °C with a vacuum drying oven to a constant weight, the percentage of adsorbed water for the hydrogel beads containing 0, 20, 40, 80, 120 wt% Tan is 94.56, 93.88, 93.56, 93.12, 92.33%. 

### 4.3. Characterization

Morphology of the obtained beads were revealed by a scanning electron microscopy under 10 kV (SEM, VEGA-3, Tescan, Czech Republic). The structure of the beads was demonstrated with Fourier transform infrared (FTIR, Avatar 360 Nicolet instrument). The stability of hydrogel beads was investigated in water under 50 °C, with a bath shaker at 200 rpm. The thermogravimetry (TG) analysis was conducted via a TG 209F3 instrument (NETZSCH Scientific Instruments Ltd., Shanghai, China) under N_2_ atmosphere with a heating rate of 10 °C/min^−1^. The absorbance of the MB solution was tested with a S 3100 spectrophotometer (Mapada Instruments Co. Ltd., Shanghai, China). 

### 4.4. Adsorption Experiments

The adsorption of MB from aqueous solution onto Tan/SA beads were systematically evaluated. Then, 20 mg dried Tan/SA beads were added into 30 mL of MB solution, and the solution placed in an isothermal water bath shaker at 150 rpm. The pH of the solution was adjusted with 0.1 M HCl and NaOH solution. The concentration of MB was determined by using a UV-vis spectroscopy at 664 nm. The adsorption capacity Q (mg/g) and removal efficiency R (%) were calculated as follows:

*Q* = (*C_0_* − *C_t_*)*V*/*m* (7)

*R* = (*C_0_* − *C_t_*) × 100/*C*_0_ (8)

*C*_0_ (mg/L) and *C_t_* (mg/L) are the initial concentration and remaining concentration of MB, m (g) is the weight of Tan/SA beads and V (L) is the volume of the solution.

## Figures and Tables

**Table 1:** Thermodynamic parameters for the adsorption of MB on 40% Tan/SA beads.

Temperature (K)	*K* ^0^	Δ*G*^0^ (kJ/mol)	Δ*H*^0^ (kJ/mol)	Δ*S*^0^ (kJ/mol·k)
288	0.36	2.44	80.80	0.27
298	0.69	0.92	-	-
308	2.53	−2.38	-	-
318	8.25	−5.58	-	-

**Table 2:** Kinetic parameters of three models for MB adsorption.

Kinetic Models	Coefficients	60 mg/L	80 mg/L	100 mg/L
Pseudo-first-order	*q*_e,cal_ (mg/g)	9.7279	15.8790	27.4949
	*k*_1_ (min^−^^1^)	0.0107	0.0139	0.0172
	*R* ^2^	0.9029	0.8050	0.9666
Pseudo-second-order	*q*_e,cal_ (mg/g)	86.81	116.55	144.93
	*k*_2_ (×10^−3^) (g/mg min)	2.78	2.07	1.31
	*R* ^2^	0.9999	0.9999	0.9999
Elovich model	*α* (mg/g min)	3.14 × 10^6^	5.87 × 10^6^	2.89 × 10^5^
	*β* (g/mg)	0.219	0.166	0.110
	*R* ^2^	0.8147	0.7301	0.8463

**Table 3:** Comparison of the MB removal performance with similar adsorbents.

Adsorbent	*C*_0_ (mg/L)	*Q_max_* (mg/g)	Removal (%)	Regeneration	Ref.
Tan/SA	200	247.2	82.4	>90%, 5 cycles	This work
Lig/CS	82	36.25	88.4	ND	[5]
CS/SP	300	40.986	27.3	ND	[6]
SA/SB/HNT	100	49	94	ND	[7]
Fe_3_O_4_/AC/SA	700	222.3	31.8	ND	[8]
SA/AC	1000	730	73	ND	[9]

## Data Availability

Not applicable.
